# Disseminated Tuberculosis With Cardiac Tamponade in an Immunocompetent Individual

**DOI:** 10.7759/cureus.16088

**Published:** 2021-07-01

**Authors:** Mário Bibi, Joana Monteiro, Nídia Oliveira, Marta Pereira

**Affiliations:** 1 Internal Medicine, Hospital Pedro Hispano, Porto, PRT; 2 Oncology, Instituto Português de Oncologia do Porto, Porto, PRT; 3 Intensive Medicine, Hospital Pedro Hispano, Porto, PRT

**Keywords:** disseminated tuberculosis, cardiac tamponade, constrictive pericarditis, mycobacterium tuberculosis, pericardial tuberculosis

## Abstract

We report a case of disseminated tuberculosis with cardiac tamponade in a 26-year-old man from northern Portugal. He was imprisoned for one year before the diagnosis and had no known immunosuppressing conditions. A high level of suspicion with a detailed review of risk factors and exposure history (e.g., in this case, imprisonment is a risk factor for tuberculosis) is necessary when pursuing a diagnosis of extrapulmonary tuberculosis and treatment should be started as soon as possible when life-threatening manifestations occur. We used a 12-month course of antituberculosis agents associated with steroids, in our case. The patient had a good clinical response and no signs of disease at the end of the treatment.

## Introduction

Tuberculosis is caused by Mycobacterium tuberculosis, a slow-growing mycobacterium [1]. Its most common manifestation is pulmonary tuberculosis however it can potentially affect any organ [1]. Tuberculosis is the leading cause of death worldwide from an infectious disease among adults and has been associated with poverty [2]. The disease’s incidence is highest in low-income countries and its burden is also primarily borne by the most socio-economically deprived communities. [2]. Portugal has been an oddity in southwestern Europe concerning tuberculosis incidence. Despite a diminishing incidence in recent decades, it still has more than twice the number of cases per year when compared with its neighbouring country Spain (19 cases per 100,000 population versus 9.3 cases per 100,000 population, respectively) [3,4].

## Case presentation

A 26-year-old man was admitted in our emergency department (ED) in Matosinhos, Portugal, after a syncopal episode. He was imprisoned approximately for one year before admission and had a known contact with an individual with pulmonary tuberculosis. He had no known chronic diseases except for bipolar disease and was an active tobacco smoker. He reported no history of recurrent infections, including during childhood.

The patient suffered from asthenia for six months and for the previous month had dry cough, chest pain, fever and anorexia. Several antibiotics were prescribed in previous medical appointments for presumed respiratory infections (amoxicillin, azithromycin and ceftriaxone). In the three days prior to admission, he had a more intense chest pain and dyspnoea and in the day of admission he experienced syncope. At admission he was feverish (38.5ºC), tachypnoeic (35 cpm), with type 1 respiratory failure (arterial-to-inspired oxygen (PaO2/FIO2) ratio of 233), hypotensive (80/50 mmHg), tachycardia (146 bpm) with jugular venous distention and hyperlactatemia (2.7 mg/dL).

**Figure 1:**
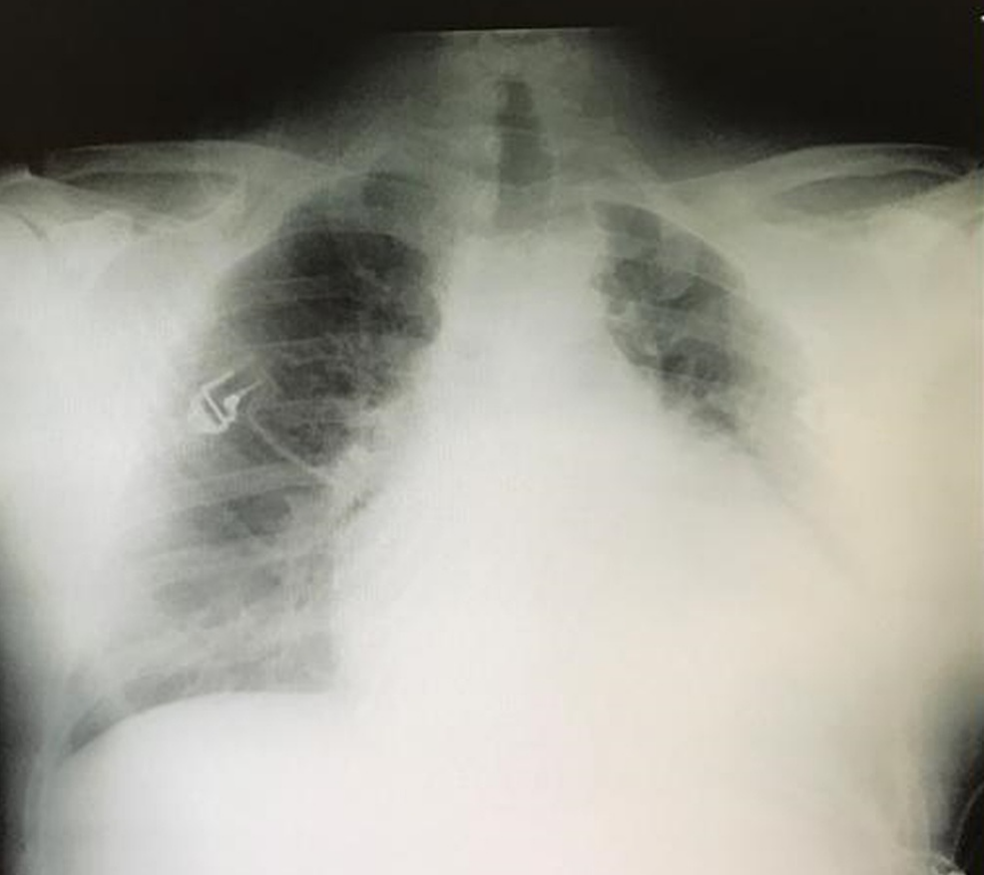
Plain radiography. Globular enlargement of the cardiac shadow giving a water bottle configuration.

**Figure 2:**
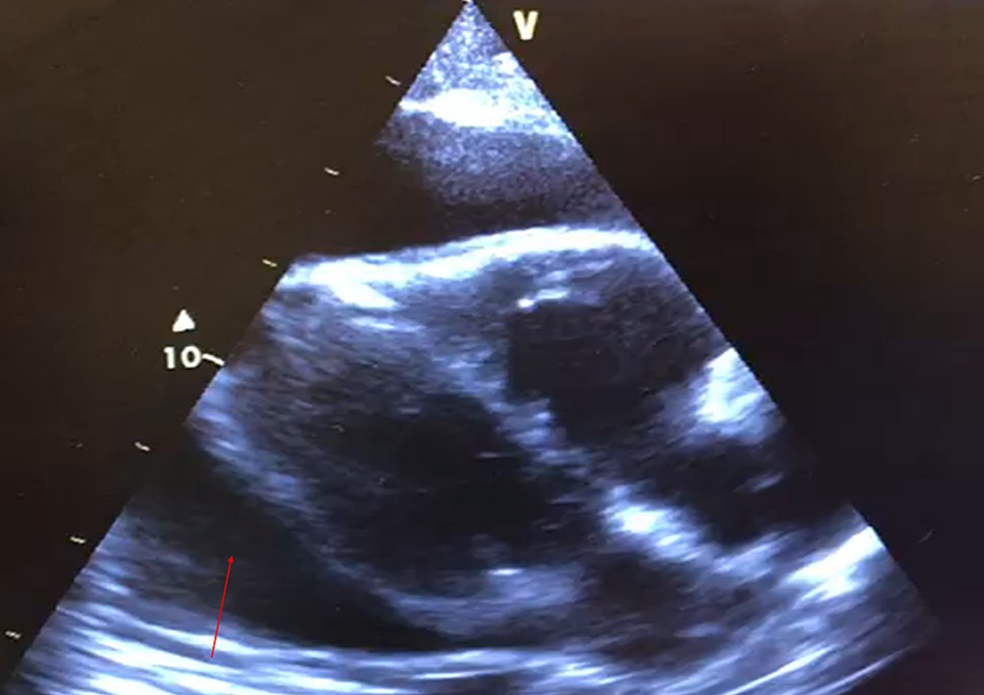
Transthoracic echocardiography. Intrinsically normal heart and a large pericardial effusion (arrow).

The plain chest radiography showed a globular enlargement of the cardiac shadow, in a water bottle configuration (Figure 1). The plain radiography from 10 days prior did not show this alteration. The transthoracic echocardiography revealed an intrinsically normal heart with a large pericardial effusion with a swinging motion of the heart within the effusion (Figure 2), with collapse of the right ventricle during ventricular diastole, and a dilated inferior vena cava due to reduced right-sided filling. A diagnosis of cardiac tamponade was made on the ED, and the pericardial fluid was drained, via echocardiography-guided pericardiocentesis, with a continuous echocardiographic monitoring. This fluid was a bloodstained exudate, with low adenosine deaminase levels (28 U/L, normal range < 40 U/L). After drainage, the echocardiogram showed signs of constrictive pericarditis.

**Figure 3:**
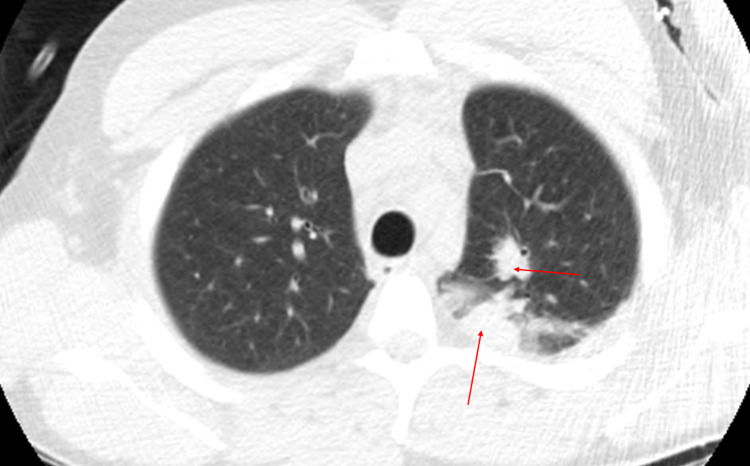
Thorax CT scan. Lung parenchyma hyperdensities in the upper left lobe (arrows).

The following investigation also found pulmonary consolidations, cervical and mediastinal adenopathies in a computed tomography (CT) of the thorax (Figure 3). An endobronchial mass was visualized by bronchoscopy. Acid-fast bacilli smear and nucleic acid amplification testing were made on several clinical specimens (sputum, bronchoalveolar lavage, pericardial fluid and tissue biopsy of endobronchial mass), all negative. Given the clinical severity and pericardial involvement, a preliminary information of necrotizing granulomas on the endobronchial mass’ biopsy led us to initiate empiric antituberculosis therapy (isoniazid 300 mg/day; rifampicin 600 mg/day, pyrazinamide 2250 mg/day, ethambutol 1600 mg/day) associated with corticosteroids (prednisolone 1 mg/kg/day).

Mycobacterium tuberculosis was eventually only identified in the mycobacterial culture of sputum and bronchoalveolar lavage specimens. A diagnosis of disseminated tuberculosis with pulmonary, pericardial and lymph node involvement was made. Antimicrobial susceptibility testing revealed no resistances to the commonly used antituberculosis drugs. No immunocompromised state was found, specifically human immunodeficiency virus serology was negative and immunoglobulins' levels and lymphocyte immunophenotyping were normal.

The patient completed twelve months of antituberculosis therapy (two months of isoniazid, rifampin, ethambutol and pyrazinamide plus 10 months of isoniazid and rifampin) and eight months of corticosteroids (with gradual weaning). He had a complete clinical response to the treatment, with symptom resolution, a clear thoracic CT scan and no pericardial effusion or constrictive pericarditis on echocardiogram one year after the diagnosis.

## Discussion

Acute bacterial infections of the lower respiratory tract can be confused with pulmonary tuberculosis, especially if a good anamnesis is not taken and risk factors for tuberculosis are not sought [5]. Our patient had chronic symptoms and had been imprisoned for one year. The augmented risk of tuberculosis among the inmate population is well characterized, and originates from the combination of prison population’s demographics (e.g. drugs users, homeless, low socioeconomic status) with vulnerabilities of the prison setting (e.g., poor ventilation, overcrowding) [6].

The gold standard method for diagnosis is culture from samples collected from the affected organ, most frequently the lung, via sputum, bronchial secretion or bronchoalveolar lavage sampling [7]. If extrapulmonary tuberculosis is suspected, other samples should be investigated such as aspirates, biopsies or body fluids (e.g., urine, stool, pericardial fluid) [7]. The culture method has a high sensitivity but it can take weeks to yield results, so other diagnostic tools should be used for a more rapid diagnosis [7]. Two methods are frequently employed: direct microscopic demonstration of the pathogen using Ziehl-Neelsen stain and nucleic acid amplification tests (NAAT) [8]. Both methods have varying sensitivities, depending on the burden of mycobacteria and on the laboratory performing the tests; however, NAAT seem to have higher overall sensitivity [8]. As seen in our case, it can be difficult to identify the mycobacteria, so in highly suspected cases, multiple samples should be taken, while excluding other diagnoses. Sometimes diagnosis can only be made after a good response to empirical treatment is obtained, especially in life-threatening situations [9]. The decision to initiate empirical treatment should be done with caution as antituberculosis treatment is associated with some serious adverse effects, notably hepatitis, and close monitoring of the patient (evaluating response to treatment and side effects) is needed [10]. A mention should be made regarding adenosine deaminase (ADA). ADA levels in pericardial or pleural effusions have been used to rapidly identify the tuberculous etiology as high level of ADA are associated with a sensitivity and specificity of around 90% [11]. However, the elderly, the critically ill with multiorgan failure and active smokers can have normal ADA levels, even in the presence of pleural tuberculosis [11]. In our case, the patient was critically ill and was an active smoker, which could explain the low ADA levels.

Disseminated tuberculosis is defined as the presence of two or more noncontiguous sites resulting from lymphohematogenous dissemination of Mycobacterium tuberculosis [12]. Extrapulmonary tuberculosis is more frequent in low-income countries but seems to be rising in high-income countries, owing in part to the emergence of the HIV/AIDS pandemic and widespread use of immunosuppressive drugs [13]. Increasing concern exists given the current refugee crisis and mass migration to western countries [14]. In Northern Portugal, 6 cases of disseminated tuberculosis were reported in 2008 and 11 cases in 2018 [15]. Treatment duration of disseminated tuberculosis should be individualized, according to clinical response, with a duration that ranges between 6 and 12 months [7]. In Portugal, current guidelines recommend 12 months [16].

Cardiac tamponade is a medical emergency that can rapidly result in death if not treated [17]. The mortality risk depends on the fast diagnosis and treatment, as well as the cause of the tamponade [17]. In developed countries, Mycobacterium tuberculosis infection rarely presents as pericardial disease (<1%), and when it does, it normally occurs in immunocompromised patients [18]. Left untreated, pericardial tuberculosis leads to a median survival time of 3.7 months [18]. Treatment involves standard anti-tuberculosis drugs with the potential addition of corticosteroids, especially in those patients with constrictive pericarditis [18,19]. Constrictive pericarditis is a feared sequelae of pericardial tuberculosis, as it can lead to chronic heart failure and significant morbidity even after successful eradication of the mycobacteria [18]. A recent retrospective study of 50 immunocompetent patients with tuberculous pericarditis found that 80% of the patients with constrictive physiology at initial diagnosis responded to treatment (antituberculosis medication and steroids) and had improved echocardiogram parameters [20].

## Conclusions

Despite the rarity of pericardial involvement by Mycobacterium tuberculosis, especially in an immunocompetent individual residing in a developed country, this diagnosis should not be overlooked if the remaining epidemiological and clinical data suggest this aetiology. Special attention should be paid to the incarcerated population. The high index of suspicion allows faster diagnosis and treatment initiation, even with initial negative diagnostic results, improving the prevention of complications, such as those arising from constrictive pericarditis.
